# Efficacy and safety of a new robot-assisted stereotactic system for radiofrequency thermocoagulation in patients with temporal lobe epilepsy

**DOI:** 10.3892/etm.2014.1620

**Published:** 2014-03-12

**Authors:** ZHAOHUI WU, QUANJUN ZHAO, ZENGMIN TIAN, JIANNING ZHANG, XIA XIAO, HONG LIN, HONG WANG, FULI WANG

**Affiliations:** Department of Neurosurgery, Navy General Hospital of People’s Liberation Army, Beijing 100048, P.R. China

**Keywords:** stereotactic techniques, robotics, deep electrodes, radiofrequency thermocoagulation, epilepsy

## Abstract

The aim of the present study was to evaluate the efficacy and safety of a newly developed robot-assisted frameless stereotactic system for deep electrode implantation and radiofrequency thermocoagulation (RFTC). Deep-electrode implantation was performed in the bilateral mesial temporal lobes of seven patients. Following the implantation of the deep electrodes through the monitored designed path, the epileptogenic zones were determined with the assistance of a robot system. Deep electrode electroencephalograms were recorded prior to and following RFTC. Treatment outcomes were evaluated by computed tomography scans and Engel classification criteria. The procedure was well tolerated by all patients with no patients suffered from severe permanent complications. After follow-ups for 34–62 months, four patients achieved Engel class I, including three patients with Ia classification, two patients were classified as Engel class IVa and one patient was classified as Engel class IVc. Therefore, robot-assisted frameless stereotaxy for deep electrode implantation and RFTC is indicated to be a safe and effective method that may be used effectively in clinical practice.

## Introduction

Invasive presurgical electroencephalogram (EEG) recordings of seizures are required to define the optimal cortical resection in patients with intractable epilepsy (1,2). Alternative neurosurgical approaches that are minimally invasive have been sought for a number of years. Recent advances in computed tomography (CT) technology have enabled physicians to safely and accurately manipulate a needle under CT fluoroscopy (3,4). Radiofrequency thermocoagulation (RFTC), under the guidance of a robot, is considered to be a safe and minimally invasive technique that may be used to treat intractable epilepsy (5,6). The methodology of deep electrode implantation was first developed by Bancaud and Talairach (7); however, the RF was not efficient in thermocoagulating the epileptic foci (8,9). The deep electrodes are fundamental to this method, as they are stereotactically implanted in the brain to identify the exact locations of the epileptogenic zones (EZs) and the pathways of seizure propagation (9). An advantage of this method is that it is possible to define epileptogenic zones (EZs) without performing a craniotomy. The deep electrodes in the mesial temporal lobe record the discharges in the hippocampus and the deep electrodes in the white matter of the frontal lobe record the discharges in the gray matter of the frontal lobe.

The robot CAS-R-2 frameless stereotactic system is a medical robot system recently developed by the Robotics Institute of Beijing University of Aeronautics & Astronautics and the Navy General Hospital (Beijing, China). The system provides accurate RF treatment of the cranium and assists in deep electrode implantation. In the present study, the efficacy and safety of this robot-assisted system was evaluated by implanting deep electrodes in the bilateral mesial temporal lobe through the frontal lobe. EZs were located in patients with temporal epilepsy and deep EEG-guided RFTC treatment was conducted, with control of the temperature and time. CT scans were used to determine whether RFTC was performed effectively and Engel classifications were assigned for evaluation.

## Patients and methods

### Patients

The study was approved and registered by the Ethics Committee of the Navy General Hospital of People’s Liberation Army in February 2008. The Ethics Committee approved associated treatment, data collection and patient follow-ups. All patients provided written informed consent and all procedures were conducted following the provisions of the Declaration of Helsinki.

A total of seven patients were selected successfully following screening (female, 4; male, 3; age, 29.6±11.1 years; age range, 16–45 years). The patients were selected according to the following inclusion criteria: i) aged ≥15 years-old; ii) experienced simple and/or complex partial seizures with or without secondary generalization; iii) experienced ≥3 complex partial seizures during the 3-month (12-week) baseline seizure diary period, with ≥1 seizure occurring within the last 2 months. and iv) electrographic evidence of seizures arising from one temporal lobe, with radiographic evidence of mesial temporal sclerosis in the same temporal lobe. Patients with normal magnetic resonance imaging scans, bilateral hippocampal damage or cortical lesions were excluded.

Since the data obtained from noninvasive presurgical investigations were not congruent for sufficiently localizing the EZs, intracerebral recordings of seizures, including video-deep EEG and positron emission tomography/CT, were performed prior to surgery. The necessary laboratory examinations were also performed, including blood cell count, blood specimen collection, alanineaminotransferase, aspartateaminotransferase, creatinine, ureanitrogen, partial thromboplastin time, prothrombin time, fibrinogen, ABO blood-group, blood grouping and crossmatching, HIV antibodies, hepatitis C antibodies and hepatitis B surface antigens.

### Implantation procedure

Two recording deep electrodes (SD06R-SP10X-000, Ad-Tech Medical Instrument Corp, Racine, WI, USA), were implanted using the robot CAS-R-2 frameless stereotactic system. Following the placement of four markers on the foreheads of the patients, MRI scans at 3 mm intervals were collected. MRI scans of the head were three-dimensionally reconstructed with a computer. Next, the placement targets of the deep electrodes were marked in the workstation. The deep electrodes were implanted through the monitored designed path (transfrontal) and avoided the important blood vessels and functional area. An assistant collaboratively calibrated the mechanical arm to a zero position. Following the registration of placement targets in the workstation, the mechanical arm was calibrated, locked in position and the direction and cranial point of craniotomy was determined.

**Figure 1:**
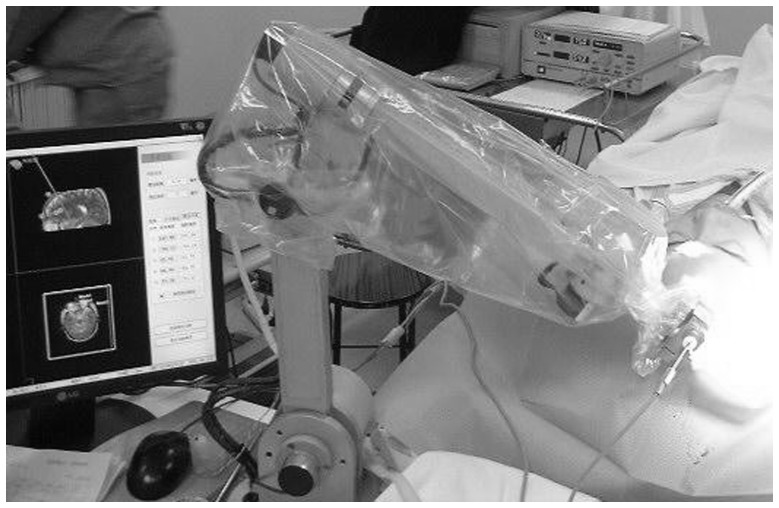
Robot-assisted stereotactic puncture surgery. The image shown on the computer screen located at the left of the figure shows the robot-manipulator auxiliary intracranial target.

Following the administration of 2 ml lidocaine (2%) for anesthesia, patients were placed in a supine position for surgery. The heads of the patients were immobilized with a molding pillow. Percutaneous sphenotresia was performed following the removal of the puncture needle to facilitate the implantation of the deep electrodes (Fig. 1). Once the electrodes had been confirmed to function normally, the wound was sutured and covered by a sterile dressing. Postoperative cranial CT scans were performed to confirm that the electrodes were positioned accurately. Patients with no intracranial bleeding complications were transferred to the EEG monitoring room.

### Monitoring procedure

**Figure 2:**
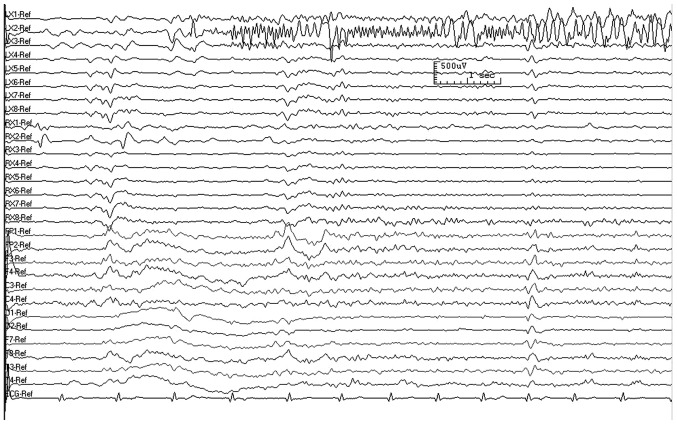
Deep electrode EEG recording of a patient with a seizure in the left medial temporal lobe. EEG, electroencephalogram.

**Figure 3:**
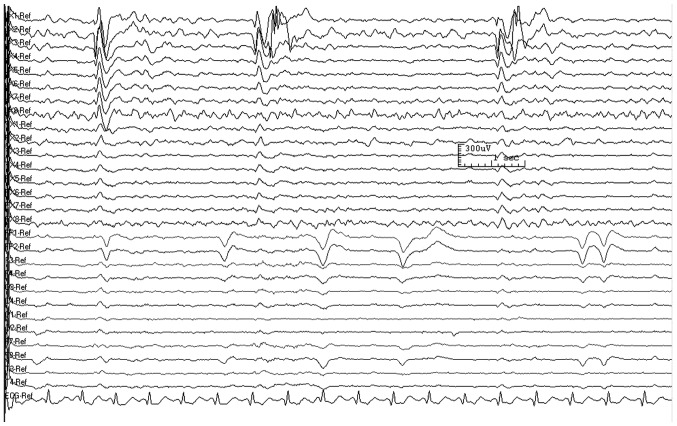
Left temporal spike wave rhythm shown in a deep electrode EEG recording following the injection of bemegride. A sharp and slow wave rhythm is exhibited. EEG, electroencephalogram.

The duration of deep electrode EEG monitoring ranged between 6 h and 7 days. At least one clinical seizure was recorded in each patient from which the EZs were determined. Fig. 2 shows a deep electrode EEG recording of a seizure in the left medial temporal lobe. If the patients had no natural seizures during the first 24 h of monitoring, 150 mg bemegride was injected intravenously at 25 mg/min. The injection was stopped immediately when the patient underwent seizure aura. Fig. 3 shows a deep electrode EEG recording following the injection of bemegride. Phenobarbital (100–150 mg) was injected immediately into patients experiencing a seizure to prevent status epilepticus.

### Robot-assisted RFTC procedure

General anesthesia was administered intravenously prior to surgery. Ventilation was controlled with an anesthesia machine and muscle relaxant was provided if necessary. Deep brain resistance measurements and 0–6 V electrical stimulation (2 Hz) were performed prior to RF. During this process, close attention was paid to whether the patient experienced limb movement, and the position of the nucleus of epileptic was confirmed. RF radiation was targeted to the amygdala firstly and the actual EZs were evaluated by intraoperative EEG. The selection of targets was determined from video-deep EEG recordings regarding the localization of the onset zone. Radiation was stopped once interictal paroxysmal activities disappeared.

An RF generator system (Radionics Medical, HXKN Ltd., China) was used to conduct RFTC in 2 or 3 mm intervals at 75°C for 120 sec. Once the epileptiform discharges disappeared from the deep electrode EEG recordings, surgery was stopped. Following compensation, if the epileptiform discharges remained, RFTC was performed in the ipsilateral or contralateral amygdala hippocampus. Deep EEG recordings were performed for ≥5 min prior to and following the RFTC procedure. Surgery was terminated once the frequency of the spike or sharp waves on the deep EEG recordings was <75% of those recorded prior to robot-assisted RFTC.

Surgery was stopped immediately following a seizure to prevent damage due to fracturing of the intracranial needles.

### Postoperative treatment and follow-up

Antiepileptic drug treatment was not changed for 6 months following RFTC, but was modified at the 6 month follow-up if required. The frequency of seizures and the possible adverse effects of the RFTC procedure were recorded during the follow-up period. During the follow-ups, patient complaints with regard to memory, attention and language were recorded. Routine tests were performed as part of the neurological evaluation and Engel classifications (1993) (10) were assigned for neurological function evaluation.

## Results

### Patients

The numbers of EZs treated, their locations and the seizure frequencies prior to and following RFTC are listed in Table I. The average disease course was 14 years (range, 2–40 years). Five patients experienced no pre-attack symptoms, one patient had fear as a seizure precursor and one patient’s seizures were preceded by a feeling of discomfort in the chest.

Patients received CT scans following deep electrode implantation surgery. All the electrodes were implanted properly to the designed locations. No permanent neurological or cognitive impairments occurred following these procedures. However, one patient had a mild subarachnoid hemorrhage (confirmed by CT scan), but following the correct treatment did not suffer from neurological function impairment. An average of 8.1±3.1 EZs (range, 6–13) were treated in one or more anatomical targets. The EZs were located in the unilateral or bilateral hippocampus and/or amygdala. The follow-up period ranged between 34 and 62 months, with a mean follow-up period of 44.6±12.2 months.

### Deep EEG results

All seven patients had interictal epileptiform discharges in the bilateral mesial temporal lobe, as shown by the deep EEG recordings. The clinical seizure onset zone of one patient was the left frontal lobe, while for the other six patients it was the unilateral or bilateral mesial temporal lobe. Due to the shortened time between the brain electrical origin following bemegride injection and the induction of an epileptic seizure, positioning errors or difficulties may have occurred (data not shown).

### Engel classification outcome

**Figure 4:**
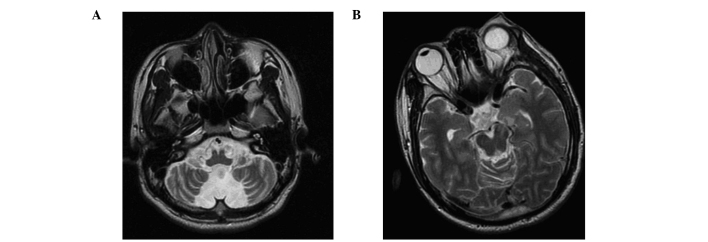
Comparison of MRI scans prior to and following robot assisted RFTC treatment. (A) Cerebellar hypoplasia was confirmed preoperatively. (B) Following robot assisted RFTC treatment, the volume of the right hippocampus still less than the left, the right temporal horn still larger than left and the target of RFTC treatment was the head of the left hippocampus. RFTC, radiofrequency thermocoagulation; MRI, magnetic resonance imaging.

Following the last consultation, four patients were classified as Engel’s class I (three patients, Ia and one patient, Id), two patients were classified as Engel class IVa and one patient was classified as class IVc. Seizure frequency decreased by ≥50% in six patients and four patients were seizure-free. Postoperative CT scans indicated that the robot-assisted RFTC treatment was safe and effective (Table I). Fig. 4 compares the pre- and postoperative CT scans of a patient who received robot-assisted RFTC treatment.

## Discussion

A deep electrode robot-assisted frameless system was first reported by Eljamel (11). Since then, a number of studies have been performed with the aim of further improving this system (12–14). The main characteristics of deep electrode implantation are firstly that the electrodes are implanted by a stereotaxic method alone, instead of using strip or grid electrodes. In addition, epileptiform discharges are evaluated by space and time, and then the cortical origin, form of communication and the focal cortical areas involved are identified. In the present study, the deep electrodes were implanted with the assistance of a robot. CT scans showed that the implanted positions were consistent with the preoperative planning. This preformation shortened the surgery time, minimized pain and increased the precision. Furthermore, the procedure was acceptable for the majority of patients in the Navy General Hospital.

The CAS-R-2 robot-assisted frameless system was designed by the Robotics Institute of Beijing University of Aeronautics & Astronautics and the Navy General Hospital of People’s Liberation Army. The system, without RFTC, has been applied successfully in clinical practice for neural stem cell therapy and, consistent with the results of the present study, achieved satisfactory curative effects.

During the deep EEG recording process, the presence of electrodecremental events, high frequency activity, irregular sharp waves intermixed with slow activity, spike-wave activity and rhythmic ictal transformation at the seizure onset determined the onset zones of partial complex seizures (15). Park *et al* classified seizure onset patterns as rhythmic activity, attenuation, repetitive spikes or spike-wave complexes, and indicated that the common pattern of seizure onset was rhythmic activity and repetitive spikes (16). The authors classified focal or regional seizure onset based on the number of electrode contacts that were involved in the ictal EEG and found that temporal lobectomy lead to excellent outcomes for focal seizure onset. Assisted by the robot system, the onset zones in the unilateral or bilateral mesial temporal lobes were defined in all seven patients in the current study. The discharges of the frontal lobe were recorded by the electrodes in the white matter since the deep electrodes were implanted in the planned targets properly. During the complete robot-assisted RFTC process, complications were minor and no long-term side-effects were observed. Guénot *et al* (17) performed stereotactic EEGs for 100 cases, and in each case, 5–15 (average, 11) deep electrodes were implanted. Complications occurred in five cases with two patients suffering from a skin infection, two patients with electrode fracture and one patient who succumbed to an intracranial hematoma. These results demonstrate the safety of stereotactic EEG.

The application of stereo implantation in the present study mainly focused on distinguishing the different lobe of epilepsy in the frontal and temporal sides. The dorsolateral prefrontal approach was adopted in the current study. The appropriate angle of the deep electrode was adjusted and epileptiform discharges in the white matter of the frontal lobe were recorded by deep electrodes. Discharge in the white matter of the frontal lobes has auxiliary effects on determining the origin of epilepsy. If properly applied, deep electrode EEG has the clinical diagnostic value of a cortex electrode (18).

The RFTC procedure on unilateral or bilateral mesial temporal lobes was particularly favorable for patients whose epileptic onset zone was the mesial temporal lobe. In the present study, six of the seven patients benefited from RFTC with a reduction of ≥50% in seizure frequency. The results obtained compare favorably with those of other palliative therapeutic procedures, including vagus nerve stimulation, multiple subpial transaction, callosotomy or deep intracerebral stimulation (19). Reported Engel I classification rates were >70% (18,20), but the effect varies in patients with different indications (21,22). In the current study, robot-assisted RFTC was performed in patients with intractable epilepsy. Discharges existed in the frontal and temporal interictal epileptiforms and deep electrode EEG recordings showed that the medial temporal lobe was the seizure onset zone. Transfrontal RFTC was performed in the deep temporal lobe structure using the stereotactic system robot. The proportion of patients with an Engel classification of I was 57% (4/7). However, further studies are required to confirm these results.

In conclusion, the present study demonstrated that robot-assisted RFTC is effective for patients with intractable epilepsy; the key lies in the identification of the correct epileptic locations. The robot-assisted stereotactic system is capable of performing procedures with deep electrodes and RF and demonstrates a good example of the transformation of engineering technology to a medical application.

## Figures and Tables

**Table I:** Treatment outcomes of seven patients who received robot-assisted RFTC.

Coagulations	RFTC targets	Follow-up time, months	Seizure frequency prior to RFTC, per month	Seizure frequency after RFTC, per month
4R/7L	Bilateral hippocampus	62	10	1
8L	Left amygdala and hippocampus	62	4	4.5
6L	Left hippocampus	43	10	0
8L	Left amygdala and hippocampus	38	6	0
5R/5L	Bilateral hippocampus	38	10	5
6L	Left hippocampus	35	2	0
6R/7L	Bilateral hippocampus	34	2.5	1

nR, number of coagulations on the left; nR, number of coagulations on the right. RFTC, radiofrequency thermocoagulation.
